# Deep Learning Neural Network Performance on NDT Digital X-ray Radiography Images: Analyzing the Impact of Image Quality Parameters—An Experimental Study

**DOI:** 10.3390/s23094324

**Published:** 2023-04-27

**Authors:** Bata Hena, Ziang Wei, Clemente Ibarra Castanedo, Xavier Maldague

**Affiliations:** 1 Department of Electrical and Computer Engineering, Université Laval, Quebec City, QC G1V 0A6, Canada; bata-nkirda.hena.1@ulaval.ca (B.H.);; 2 Computer Vision and Systems Laboratory, Department of Electrical and Computer Engineering, 1065, Ave de la Médecine, Université Laval, Quebec City, QC G1V 0A6, Canada; 3 School of Engineering, University of Applied Sciences in Saarbrücken, 66117 Saarbrücken, Germany; 4 Fraunhofer Institute for Nondestructive Testing IZFP, 66123 Saarbrücken, Germany

**Keywords:** non-destructive testing, deep learning, automated defect recognition (ADR), semantic segmentation, digital X-ray radiography

## Abstract

In response to the growing inspection demand exerted by process automation in component manufacturing, non-destructive testing (NDT) continues to explore automated approaches that utilize deep-learning algorithms for defect identification, including within digital X-ray radiography images. This necessitates a thorough understanding of the implication of image quality parameters on the performance of these deep-learning models. This study investigated the influence of two image-quality parameters, namely signal-to-noise ratio (SNR) and contrast-to-noise ratio (CNR), on the performance of a U-net deep-learning semantic segmentation model. Input images were acquired with varying combinations of exposure factors, such as kilovoltage, milli-ampere, and exposure time, which altered the resultant radiographic image quality. The data were sorted into five different datasets according to their measured SNR and CNR values. The deep-learning model was trained five distinct times, utilizing a unique dataset for each training session. Training the model with high CNR values yielded an intersection-over-union (IoU) metric of 0.9594 on test data of the same category but dropped to 0.5875 when tested on lower CNR test data. The result of this study emphasizes the importance of achieving a balance in training dataset according to the investigated quality parameters in order to enhance the performance of deep-learning segmentation models for NDT digital X-ray radiography applications.

## 1. Introduction

Non-destructive testing (NDT) involves a vast range of inspection methods used to assess the quality and integrity of materials, components, and structures without causing damage or compromising their functionality [1]. Through the adequate application of NDT methods on components under test, flaws are detected and evaluated to ascertain if they constitute a defect that could impact a component’s fitness-for-use [2]. A wide range of industries, including aerospace, automotive, construction, manufacturing, etc., employ various NDT methods, such as visual testing, ultrasonic testing, magnetic particle testing, liquid penetrant testing, eddy current testing, and radiographic testing, which this study directly impacts. Each approach has advantages and disadvantages, and the selection of a particular NDT method is contingent on the nature of the object being inspected, the type of defect to be identified, and other relevant variables [3]. NDT X-ray radiography stands out as one of the most extensively used non-destructive testing methods [4], and this study intended to explore the rapidly evolving practices of carrying out automated defect recognition in NDT digital radiography by using deep learning algorithms.

Essentially, NDT radiography utilizes ionizing radiation to acquire images of a component’s internal structure, and it is largely employed for the inspection of welds, castings, and other structures in order to identify flaws such as cracks, voids, porosity, inclusions, and other discontinuities [5]. During NDT radiography procedures, high-energy radiation from a radiation source (e.g., X-ray tubes) is transmitted through the component under test. Depending on the density and thickness of the component under test, the incident X-ray photons experience varying levels of attenuation as they transmit, and they could also be scattered due to the interactions with atoms. If a component has flaws or changes in material properties or thickness, the incident X-ray photons are differentially attenuated by the component as they transmit through. The transmitted X-ray photons then have a latent pattern which is sensed by radiographic film or a digital detector, and then converted to a two-dimensional radiographic image of the component. Hence, NDT radiography images could provide significant information about an object’s internal structure, and can reveal internal flaws, counting as one of its advantages over many other NDT methods. Conventionally, the practice of NDT radiography is conducted by qualified NDT technicians, using specialized equipment and methods. These qualified NDT technicians should have undergone relevant trainings and are qualified and certified to perform NDT radiography according to norms such as ISO 9712 [6], ASNT SNT-TC-1A [7], etc. In addition, they should demonstrate the ability to keep to relevant NDT Standards that govern the processes involved in the acquisition and evaluation of radiographic images.

With the increasing proliferation and adoption of process automation in the manufacturing industry, high manufacturing throughput of components is achievable [8,9,10,11]. The need to inspect these fabricated components places a huge demand on NDT technicians [12], who traditionally employ trained skills and experience to manipulate X-ray acquisition setups, and visually assess and evaluate NDT radiography images. This evolving scenario birthed the need for NDT radiography to adopt non-conventional approaches in performing radiographic inspections [13] in order to ameliorate the shortage in the number of qualified NDT radiography personnel available to carry out such arduous inspection tasks, and reduce the occurrence of human error [14]. Therefore, numerous industries have incorporated automated NDT radiography systems to carry out image acquisitions more swiftly and effectively, thereby increasing the inspection throughput. These systems often use robotic arms to position the component to be tested between the radiation source and detector, thereby considerably reducing the need for manual manipulations. Nevertheless, the use of such automated systems comes with associated challenges [15], as these systems are expensive to purchase, need specialized training to use and maintain, and necessitate more sophisticated calibration and validation procedures to ensure accuracy and precision of the systems.

### 1.1. Automated Defect Recognition (ADR) in Digital NDT Radiography

The automation of image acquisition in NDT digital radiography [11,16] is only one piece of the puzzle. To ensure that proper decision-making regarding the reliability of a tested part is achieved, it is essential to assess the acquired images for quality in accordance with operational NDT Standards, identify relevant indications (flaws), and evaluate if the detected flaws constitute a defect or not (see ASTM E1316-17a, Standard Terminology for Nondestructive Examinations) [2]. To automate this other piece of the puzzle, researchers have developed Automatic Defect Recognition (ADR) for NDT digital radiography, and these solutions aim to enhance the detection and evaluation of flaws in the acquired digital radiographs of manufactured components by using different deep-learning algorithms [17]. In recent years, the prevalence of ADR systems in NDT radiography has significantly increased, gaining recognition in the industry and research [18,19,20,21]. If adequately trained, ADR solutions based on deep-learning algorithms could assess radiographic images and automatically detect flaws, thereby increasing its potential to improve flaw detection accuracy, decrease the likelihood of human error in image evaluation, and increase image evaluation throughput. Nevertheless, there are possible risks associated with the use of ADR approaches in non-destructive testing radiography. Of significant concern is the potential for ADR to produce false-positive or false-negative results. False positives arise when the ADR algorithm incorrectly finds a defect, resulting in needless repairs or component rejection. When the ADR algorithm fails to detect a flaw that exists, this is known as a false negative, and it could result in a potential safety hazard or component failure during service [22,23]. A further concern is the possibility that an ADR algorithm trained on a given set of flaw types and sizes could fail to detect flaws that do not belong within this set—a situation referred to as non-generalization of the model. This could cause the ADR algorithm to miss flaws, thus posing a safety concern. To solve these issues, it is essential to carefully train and evaluate the ADR algorithm to ensure its optimal performance.

The presence of industry-wide standardization and regulation of the development of ADR solutions remains very subtle, as known bodies that offer regulatory oversight on the practice of NDT radiography are only recently coming up with guidelines for the adoption of ADR solutions in radiographic images [24]. There is a need for standardization of such solutions, as a variety of ADR solutions with varied algorithms and capabilities are offered from numerous developers, some of which may lack the awareness of the expectations according to given NDT Standards that have overseen the practice of NDT digital radiography image evaluation in the industry for many decades.

### 1.2. Objectives of the Study

This experimental study aimed to evaluate the influence of two important radiographic image quality parameters, namely signal-to-noise ratio (SNR) and contrast-to-noise ratio (CNR), on the performance of Automated Defect Recognition (ADR) models in non-destructive testing (NDT) digital X-ray radiography. The outcome of this study should offer an informed basis for decision-making when preparing ADR training datasets for digital X-ray radiography, enable an understanding of the implications of these parameters, and ultimately allow for the utilization of this information to improve the generalization of ADR models. This study could potentially reduce the occurrence of false-positive and false-negative results, especially in NDT digital X-ray radiography applications.

## 2. Radiographic Image Quality

Image quality parameters are relevant in enhancing the perceptibility of flaws in NDT digital radiography [25]. It is a recommended practice that the quality of a radiographic image be optimal before conducting its assessment [26]. Several national and international standards organizations offer a guide on the determination of image quality, amongst which are standardization organizations such as the American Society for Testing and Materials (ASTM), American Society of Mechanical Engineers (ASME), International Organization for Standardization (ISO), and European Committee for Standardization (CEN).

Numerous factors influence the quality of images produced by digital X-ray radiography techniques. Notable amongst these factors are the setup used, the energy level (kilovoltage kV) [27,28], the tube current (mA), the exposure time [29], and the focal spot size of the X-ray tube used for acquisition [30]. Additionally, the detector properties, the calibration procedure, and the material properties of the inspected component could also affect the quality of acquired images. Since the interpretations of acquired radiographs have been traditionally carried out by human inspectors for nearly a century, studies have been conducted to establish the perceptibility of flaws within a radiographic image [31]. Image quality indicators (IQIs) are special devicesplaced on the object under test (usually on the source side of the object) during radiographic exposure. The appearance of the IQIs on the resultant radiographs is used to establish the radiographic image quality [32,33] and thereby ensure reliability of the setup used in detecting flaws.

A phenomenon that has a crucial influence on the quality of radiographic images is noise [4,25], which occurs at various stages in the image acquisition process, including within the X-ray source during X-ray generation, the object under test, and the digital detector array used for image acquisition. However, the main causal factor for noise is often the quantum noise of the radiation source [34]. The quantum noise results from the statistical nature of the X-ray photons generated by the X-ray source and the interaction of the photons with the detector. The exposure time is one of the primary factors that can be controlled to manage quantum noise levels, where a longer exposure time typically yields a larger number of photons being detected, thereby enhancing image quality by increasing the detectable signal and reducing the relative contribution of quantum noise [35]. Other factors that can cause noise in radiographic images include the exposure setup used, the inherent detector noise, the scatter radiation, etc. Higher noise levels could affect an observer’s ability to distinguish between a flaw and background.

### How Exposure Factors Affect Image Quality

This study employed the use of X-rays which are generated by the bombarding a positively charged high-density metal target (anode of the X-ray tube) with high-energy negatively charged electrons from the cathode of the X-ray tube [36]. This process is referred to as “bremsstrahlung” or “braking radiation,” as the electrons are rapidly decelerated by the target’s atoms, causing them to emit X-rays [37]. This sequence of events happens within the X-ray tube, and the beam of generated X-rays exits the tube through the exit port of the tube to be used for radiographic imaging. The intensity of the generated X-rays decreases exponentially as it passes thorough air, obeying the inverse square law, which states that the intensity of a given radiation is inversely proportional to the square of the distance from the source [38]. Hence, acquiring an X-ray image of the exact same component at different source-to-detector distances (SDDs) will yield varying intensity values. As the X-rays transmit through a part under test, the incident X-ray intensity is attenuated, as mathematically represented in Equation (1).

(1) $$I=I_{0}e^{-\mu t}$$

The intensity of photons transmitted across a distance is represented by *I*, while *I*_0_ stands for initial intensity of photons. The linear attenuation coefficient and the distance traverse are represented by *µ* and *t*, respectively.

During image acquisition, the X-ray photons that reach the imaging receptor, e.g., the digital detector array (DDA) used in this study, are sensed by the pixels of the DDA and then are converted to gray values that can be digitally processed and visualized [39]. It is important to mention that the equation above, which follows the Lambert–Beer exponential law could only be realized with a monochromatic X-ray source, e.g., synchrotron [40]. With the polychromatic nature of the X-ray tube used more commonly for image acquisition in NDT radiography applications, the generated X-rays have different energy levels, and this affects the intensity distributions during image acquisition [41].

The influence of each component of exposure factors that were used in radiographic image acquisition is described below:

1. X-ray tube voltage (kV): This exposure parameter is important, as it determines the energy of the X-ray photons being produced [42]. An increase in the kV value invariably leads to more X-ray transmission through a part under test, unto the detector. Consequently, this results in an increase in the SNR of the resultant image. However, high levels of kV exposure could lead to a reduction in the differential attenuation of the X-ray photons by regions of the part under test with differing thicknesses, thus reducing the CNR between a feature and background [43].
2. X-ray tube current (mA): The quantity of electrons generated by the cathode filament in the cathode assembly of the X-ray tube is determined by the tube current [44]. During X-ray generation, these electrons collide with the anode target of the X-ray tube to generate X-rays photons. Increasing the mA value will result in an increase in the number of X-ray photons generated. Hence, an increase will essentially have a greater impact in the reduction of noise and increase the signal-to-noise ratio (SNR) of the acquired image [36].
3. Exposure time (s): The exposure time in NDT X-ray procedures determines the duration for which the X-ray tube emits radiation to produce a radiograph of the object under test. The exposure time is adjusted depending on factors such as the material density, the thickness, the X-ray source’s output, and the image quality desired, and it is synchronized with the DDA’s integration time [45].

## 3. Materials and Methods

### 3.1. Phantom Aluminum Plates

**Figure 1:**
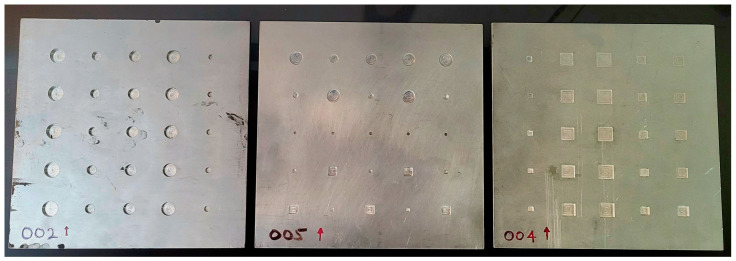
Samples from 7 aluminum plates that were used in this study, showing varying depths and shapes of drilled flat-bottom holes. Each plate measures 300 mm × 300 mm × 6.5 mm.

In this study, 7 square-shaped aluminum plates, each of dimensions 300 mm × 300 mm × 6.5 mm, were used for data acquisition. Each plate has 25 flat-bottom holes (totaling 175 for the entire 7 plates), with either circular or square shapes, and has depths ranging from 0.5 mm (shallowest) to 5.5 mm (deepest). See Figure 1.

### 3.2. Data Acquisition

A digital X-ray radiography imaging system with a maximum tube voltage of 150 kV and maximum current of 0.5 mA was used in this study. The detector is a scintillation-based 2D digital detector array (DDA) with 3098 × 3097 pixels. For the entire image acquisitions of the 7 plates, a fixed SDD of 600 mm, with the plates placed directly on the detector, is maintained. This is to ensure that, for a particular exposure factor used, consistency is maintained in gray value distribution across all plates, at regions with the same thickness. For the flat-bottom holes with varying depths, the gray values vary, forming features that can be visually appreciated in radiographs. As shown in Table 1, twenty distinct exposure parameters were used on each plate, while maintaining the same positioning, throughout the sequence of 20 exposures. This would facilitate easier annotation of features to use as ground truth for deep-learning model training.

**Figure 2:**
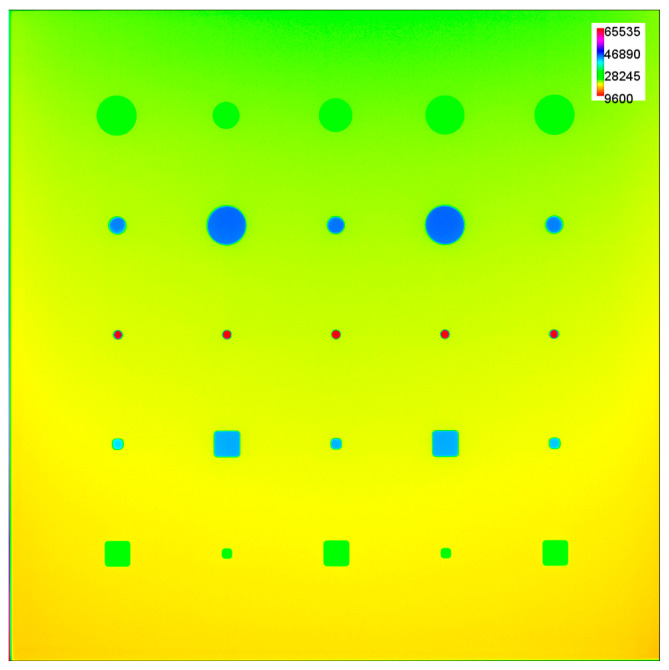
Color-spectrum conversion of the gray-value X-ray image, showing inhomogeneous intensity distribution across the 300 mm × 300 mm × 6.5 mm aluminum plate acquired with 60 kV, 400 µA, 0.5 s, at 600 mm source-to-detector distance (SDD). The depths of the drilled features on the actual plate according to the rows (top to bottom) are 1.5 mm, 4 mm, 5.5 mm, 4 mm, and 1.5 mm.

Given the DDA’s large size, a single image acquisition is sufficient to cover a 300 mm × 300 mm plate; however, the diverging nature of an X-ray beam as it travels from the focal spot of the X-ray tube to the detector results in an uneven distribution of X-ray intensity across the length and breadth of the detector. As a result, acquired images are not homogeneous in the distribution of gray values. Another factor, known as the anode heel effect, also contributes to inhomogeneity in X-ray beam intensity, due to the angled nature of the anode target of the X-ray tube, which results in the generation of X-rays with higher intensity at the cathode side, compared to the anode side, of the X-ray tube [46]. For better visualization of these effects, the inhomogeneous gray value distribution of an acquired image was converted to a color spectrum, as shown in Figure 2.

Due to the inhomogeneous GV distribution discussed above and observed in Figure 2, SNR measurements across the plates show varying values, even at regions that are of the same thickness but located at different regions. Even though this inhomogeneity was improved by the flat-fielding technique, using ISee! Software version ic-v1.11.1 [47], the effect is still noticeable in the images. The exposure factors and SNR measurements across a plate within each exposure category are listed in Table 1.

#### 3.2.1. Cropping and Dataset Preparation

**Figure 3:**
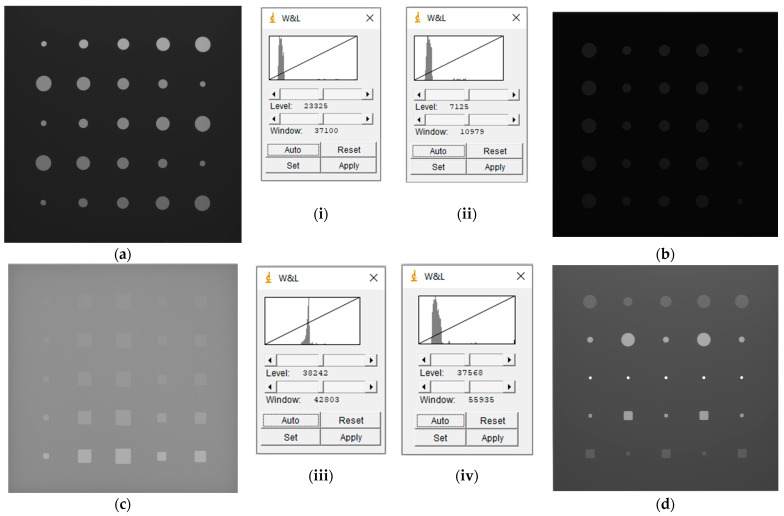
A cross-section of radiographic images of plates captured at different exposure factors: (**a**) 60 kV_200 µA_0.5 s, (**b**) 70 kV_100 µA_0.2 s, (**c**) 150 kV_50 µA_0.5 s, and (**d**) 60 kV_400 µA_0.5 s. All 16-bit images show standard gray values, with no post-acquisition image enhancement. Using ImageJ version 1.53t [48], the window and level displayed in images (**i**–**iv**) represent (**a**–**d**), respectively.

To address the limitation presented by the inhomogeneous distribution of grayscale values which inadvertently affects the SNR values across regions of the plates (as observed in Figure 2 and Table 1), we cropped each radiographic image (see examples in Figure 3) into 512 × 512 pixel regions of interest (ROIs), each containing one flat-bottom hole. Therefore, from a single image, 25 cropped images were obtained, yielding a total of 3500 images, considering 25 × 20 × 7 (feature per plate × number of exposures × number of plates).

#### 3.2.2. Data Cleaning and Selection

Given the differing depths of the flat-bottom holes, regions with holes of higher depth (lower thickness of aluminum) attained detector saturation at high exposure factors, resulting in a pixel value of 65,535 (see Figure 2). As these images could potentially have a negative impact on model training, we identified and excluded them from the dataset. With this technique, a total of 2726 candidate images without any saturated pixels were realized.

#### 3.2.3. Data Sorting

To achieve the objective of this study, the dataset containing 2726 cleaned images was duplicated. The first dataset was realized by sorting the images based on increasing SNR measurement values. This did not take into account the CNR values between the features in each image and its background. Additionally, the second dataset was sorted to increase CNR values between the feature in each image and its associated background. Like the approach with sorting using SNR, this CNR sorting operation had no consideration for the SNR values.

The signal-to-noise ratio (SNR) is the ratio of mean value of the linearized gray values to the standard deviation of the linearized gray values (noise) in each region of interest in a digital image. The NDT Standard recommends that the region of interest contain at least 1100 pixels. The SNR values are realized in accordance with Equation (2). This measurement was carried out for all the 512 × 512 cleaned images, for splitting into groups in order of increasing SNR values, with each having a specific range, e.g., 0–50, 51–100, 101–150, …, etc.

(2) $$\mathrm{SNR}=\frac{\mu_{signal}}{\sigma_{noise}}\;$$

where *μ_signal_* is the mean of the signal, and *σ_noise_* is the standard deviation of the noise.

A copy of the dataset was sorted according to contrast-to-noise ratio (CNR) values. According to EN ISO 17636-2:2022 [49], the CNR is the ratio of the difference of the mean signal levels between two ROIs to the averaged standard deviation of the signal levels. Hence, to realize this for each image in the dataset, a strategy of defining two ROIs (one on a flat-bottom hole, and the other on the background) was developed, considering the varying sizes of the features. Iterating this operation on all the images, CNR values were obtained according to Equation (3), and the dataset was categorized into different groupings, based on the realized CNR values:

(3) $$\mathrm{CNR}=\left|\mu_{1}-\mu_{2}\right|/(\frac{\sigma_{1}+\sigma_{2}}{2})$$

Mean pixel values of the feature and background ROIs, respectively, are denoted by *μ*_1_ and *μ*_2_, while the standard deviations of the pixel values of the feature and background ROIs are represented by *σ*_1_ and *σ*_2_, respectively.

**Figure 4:**
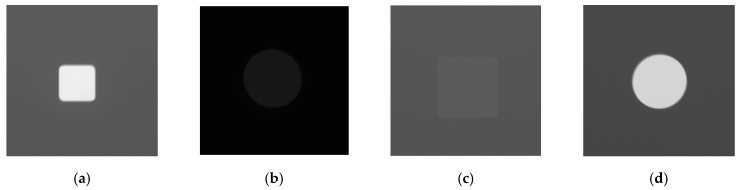

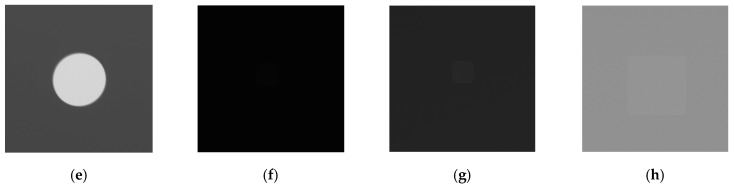
The 512 × 512 cropped images, with different image qualities resulting from the different exposure conditions used and the depth of the flat-bottom holes in each region of interest. The reduction in inhomogeneity in gray value distribution in the background could be observed in images (**a**–**h**).

A cross section of images that were methodically sorted as described above are presented in Figure 4 below, where the differing visibility of the features (flat-bottom holes) due to the depth and exposure conditions can be observed.

**Figure 5:**
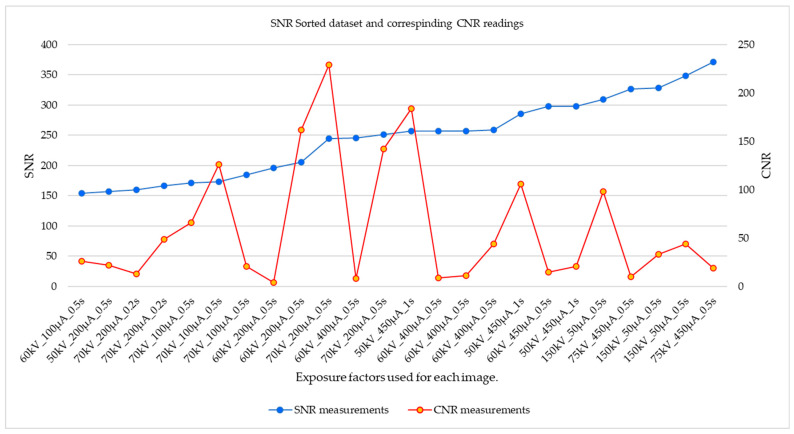
A cross-section of randomly selected data sorted in order of increasing SNR values, showing corresponding CNR values between the flat-bottom holes and background.

From the graphical plot of CNR and SNR readings for a cross-section of randomly selected cropped images shown in Figure 5, the stochastic distribution of CNR values across the dataset when sorted according to the increasing values of SNR measurements can be observed. This distribution is a function of the differing depths of the flat-bottom holes, and the exposure factors used during image acquisition.

#### 3.2.4. Data Splitting and Ground Truth Generation

To adequately explore the effect of CNR and SNR on the training of flaw-detection algorithms for NDT radiography images, the previously sorted images were classified into four distinct datasets according to their CNR and SNR values. For both datasets categorized based on CNR and SNR measurements, a spectrum of values was established and designated as high or low for each group (CNR and SNR), as can be seen in Table 2. For the four datasets realized from this, the training, validation, and test_1_ data belonged to either a high or low measurement value range of CNR or SNR. Furthermore, for each of the 4 groups, a second test dataset (*test_2_*) was realized from images that did not fall within the range of measurement values. This was to assess the impact of lacking such specific range of measured values (CNR or SNR) contained in *test*_2_, within the training dataset. The unique purpose of each subset is described below:

Training subset: This subset of the dataset holds the highest number of images (60% of the dataset) and is used to train the U-net deep neural network model. An optimization process is performed by the model, as the model learns to recognize labelled features (flat-bottom holes) on the radiographs and relationships in the data, by adjusting its weights and biases. The objective is to create a model that will be able to recognize similar features in images that are not included in the training dataset.

Validation subset: The validation set (20% of the dataset) is leveraged to assess the performance of the model during training and to fine-tune the model. Unlike with the training dataset, this validation subset is not used to adjust the model’s weights and biases, but rather to evaluate the model’s ability perform optimally on new data. Hyperparameters (e.g., learning rate) are adjusted using the validation data to avoid overfitting, which leads to a poor performance of the model on new data. Overfitting can be assessed by comparing the model’s performance on the training and validation sets.

test_1_ subset: After training and validation stages, the performance of the trained model is finally assessed using the test_1_ subset (20% of the dataset). This subset of image data is not utilized for either training or validation to avoid biasing in the model evaluation. Therefore, the model’s ability to perform optimally on unseen data (generalization of the model) is best assessed using this dataset.

*test*_2_ subset: In our study, a second test dataset (*test*_2_) was generated for each of the sorted datasets, where the respective *test*_2_ image data belong to the opposite end of the measured values of CNR or SNR considered for sorting the dataset, e.g., the dataset sorted based on high SNR values had very low SNR images for *test*_2_, and so on. The objective is to assess the generalization of the model on such dataset and study the impact that such a wide variation in image quality parameters (CNR or SNR) has on the performance of the model.

All the features on the plates were manually annotated, using CVAT [50], to generate the ground truth data for model training.

### 3.3. Deep-Learning Model Training

This study employed the use of a U-net deep-learning architecture that was initially designed for biomedical image segmentation tasks [51]. Since its inception, U-net deep-learning architecture has garnered significant interest in the field of research and is being utilized across different domains for semantic segmentation tasks [52,53,54,55,56].

**Figure 6:**
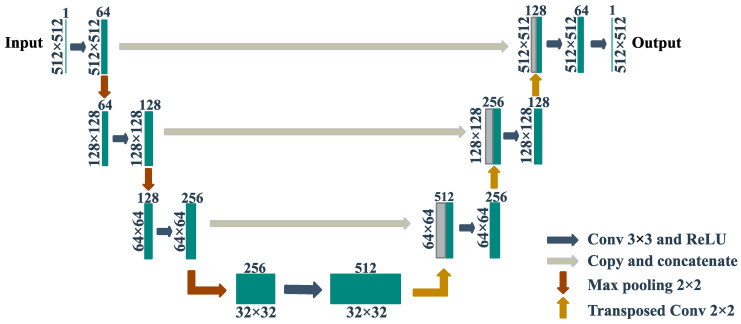
U-net deep-learning architecture.

The graphical representation of the architecture of U-net can be seen in Figure 6. The architecture comprises an encoder–decoder structure, with skip connections that enable the recovery of high-resolution features, allowing for better precision in the outcomes of segmentation tasks.

In the process of training our model, several key parameters were utilized to achieve optimal performance. The dimensions of the input images are 512 × 512 pixels, and to improve on the diversity of our dataset, we employed data augmentation techniques, including random rotation and flipping, to improve generalization. The choice of these geometric augmentation was made to maintain the quality of the images, as other augmentation techniques, such as elastic deformation, could affect the image quality parameters (CNR and SNR) being investigated. The learning rate was set to 1 × 10^−4^, and the model was trained for a total of 50 epochs. The Adam optimizer was selected to update the model’s weights, and the binary cross-entropy loss function was utilized to measure the discrepancy between the predicted output and the ground truth.

## 4. Results

The results of the model performance on the four datasets described in Table 2 are presented in Table 3 below. For each of the four datasets (High SNR, Low SNR, High CNR, and Low CNR), mean IoU values are displayed for their respective two test sets (test_1_ and *test*_2_).

**Figure 7:**
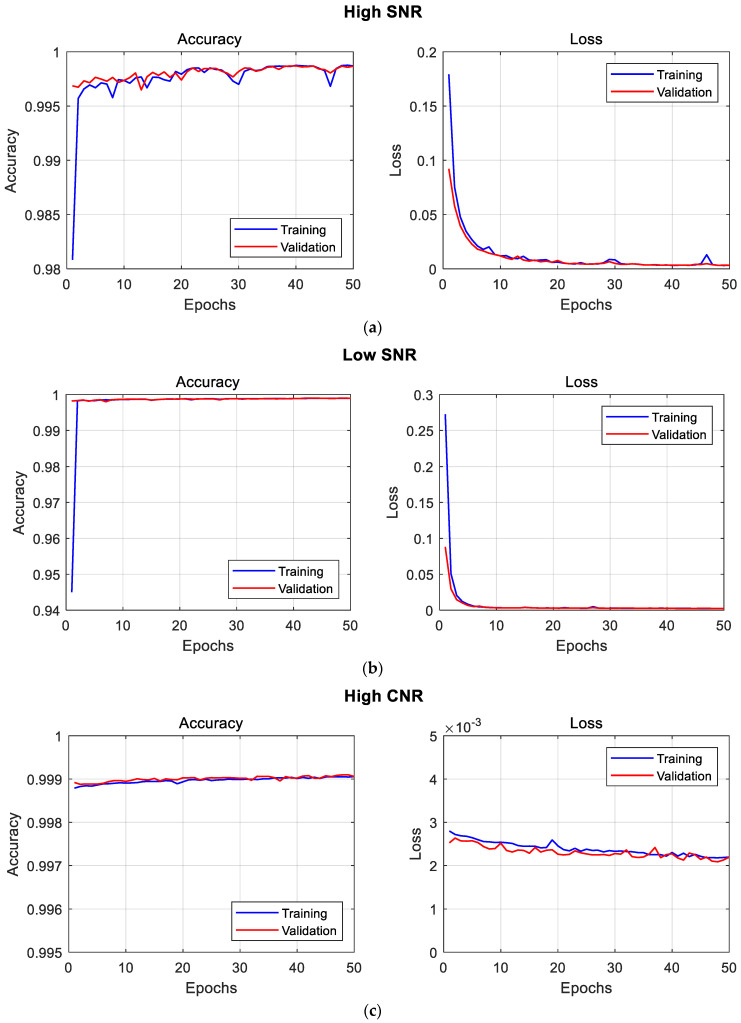

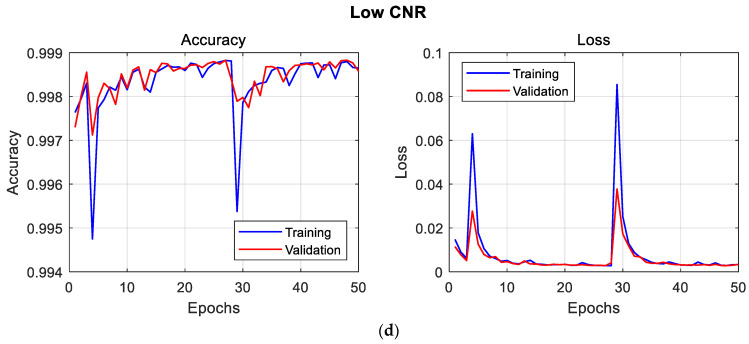
Training curves of U-net deep-learning model on High SNR dataset (**a**), Low SNR dataset (**b**), High CNR dataset (**c**), and Low CNR dataset (**d**).

For each model training session, graphical representations of the accuracy (training and validation) and loss performances during training are presented in Figure 7.

We made an interesting observation that the mean IoUs on the test images (test_1_ and *test*_2_) for the High SNR dataset were not significantly different. However, the mean IoU value of test_1_ belonging to the same category as the training datasets was slightly lower. When trained on the Low SNR dataset, a comparable model performance is observed, with the mean IoUs also having slight variations. Although, in this case, the test_1_ dataset which belongs to the same range of SNR values as the training dataset reveals a slightly better model performance.

For the High CNR and Low CNR datasets, the differences in mean IoU are comparatively higher on respective test datasets (test_1_ and *test*_2_), as shown in Table 3. As observed with the Low SNR results, the High SNR result reveals a better performance on test_1_ relative to *test*_2_. Model training with a low CNR reveals the most significant margin between its test_1_ and *test*_2_ datasets, with test_1_ yielding a lower model performance than *test*_2_, despite being in the same range of CNR values as the training and validation datasets used.

**Figure 8:**
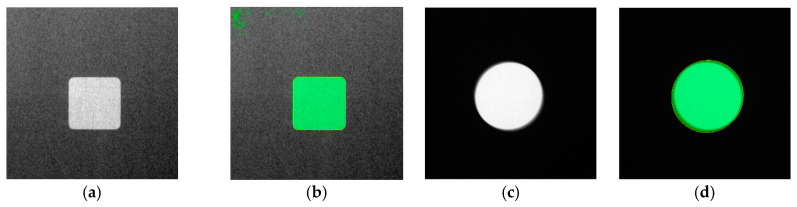

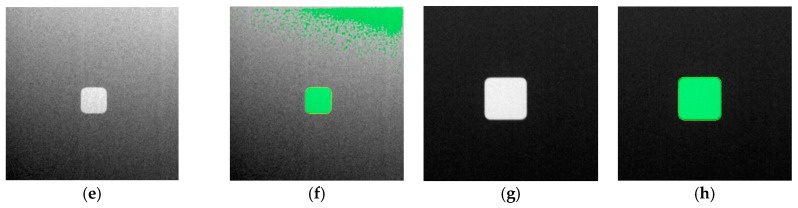
Pairs of input images and semantic segmentation results showing model performance on the training on High CNR *test*_2_ (**a**,**b**,**e**,**f**) and High SNR (**c**,**d**,**g**,**h**).

These findings, especially with the CNR datasets (see Figure 8), led to a subsequent run of model training using a different dataset, named High CNR_2_, which was sorted as performed previously (see Table 4). However, the second dataset has a narrower range of high CNR measurement values for training, validation, and test_1_, whereas the dataset for *test*_2_ has a narrower range of low CNR measurement values. This dropped the number of original images used for training.

**Figure 9:**
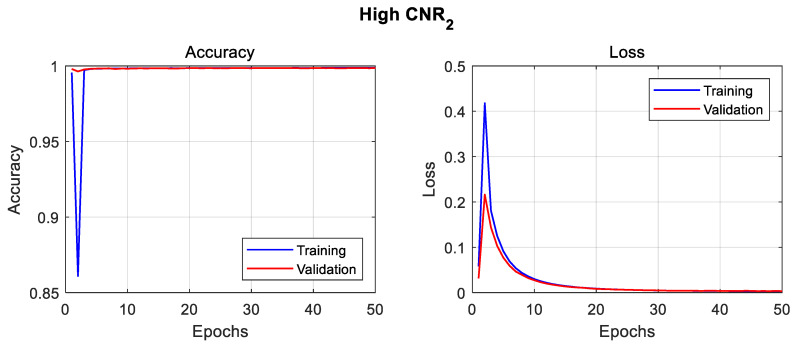
Training curves of U-net deep-learning model on High CNR_2_ dataset.

The U-net model was trained on the new high CNR_2_, while maintaining the same training parameters mentioned earlier. The results, as seen in Table 5, reveals a substantial difference between the test_1_ and *test*_2_ readings. The associated training curves can be seen in Figure 9.

## 5. Discussion

The contrast-to-noise ratio (CNR) and signal-to-noise ratio (SNR) are both associated with the signal and noise properties of a radiographic image, although they have distinct meanings. While the CNR represents the pixel intensity differences that exist between two regions of interest, the SNR addresses the overall signal quality. Hence, there is no linearized relationship between SNR and CNR readings throughout the datasets. A decrease in the CNR value could be observed in certain high-SNR images, especially when the high SNR was attained by significantly increasing the energy of the X-rays (kV) during image acquisition, leading to a reduction in the differential attenuation of X-rays between regions of different thicknesses. Therefore, sorting the data according to SNR values may inadvertently lead to a stochastic distribution of CNR values (high, mid, and low) within such a dataset as may be seen in Figure 5. The same applies to sorting according to CNR values, where the distribution of SNR values is random across the CNR-sorted dataset.

Model training on SNR datasets shows a comparable performance when tested on the respective *test*_1_ (belonging to the same SNR range as training data) and *test*_2_ (belonging to the opposite end of the measured SNR range dataset). We therefore posit that this improved performance could be a function of the diverse distribution of images with a wider range of measured CNR values across the entire SNR-sorted datasets.

**Figure 10:**
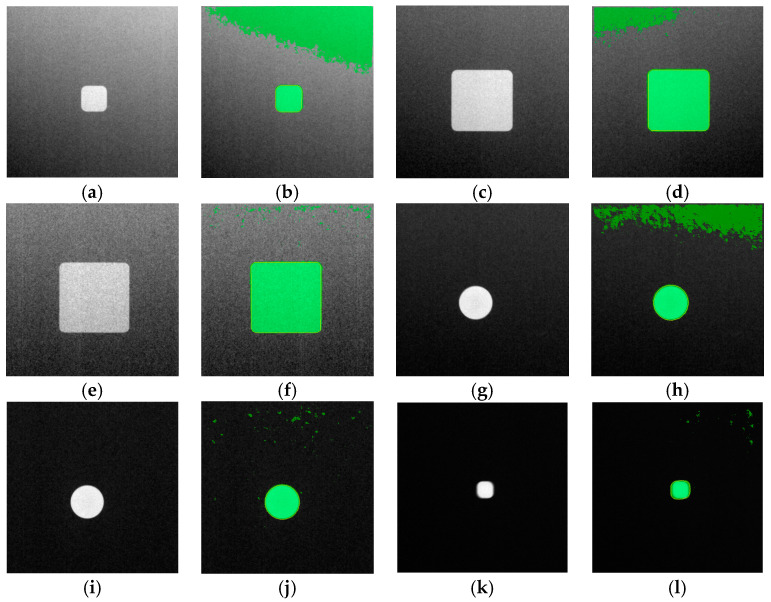
Pairs of input images and semantic segmentation results showing model performance on the training with High CNR_2_ dataset. (**a**–**l**) Pairs of image and segmentation results from *test*_2_ subset.

The subsequent findings on model performance with the High CNR_2_ dataset validate the hypothesis, where it is observed that training with datasets of a limited range of measured CNR values (at the high spectrum) performs poorly (mean IoU of 0.5875) when tested on images belonging to a limited range of low CNR values. The same trained model performed well (mean IoU of 0.9594) with test images belonging to the same range of high CNR values. Figure 10 provides a cross-section of results from this training session, where the poor performance of the trained model in segmentation is qualitatively presented.

## 6. Conclusions

The findings of this experimental study show that the image quality of digital radiography images could affect the performance of deep-learning semantic segmentation models. The contrast-to-noise ratio (CNR) emerges as the most critical image quality when compared to signal-to-noise ratio (SNR) because it focuses on specific features on the digital X-ray radiography image. The SNR remains a very important image quality parameter that is used to assess the quality of an image. According to NDT Standards, the SNR determines what testing class an acquired NDT X-ray radiographic image belongs to, causing NDT inspectors to aim toward achieving such a specific range of SNR quality. Nevertheless, it was revealed in this study that having a robust representation of flaw types at different CNR values could improve the generalization of a deep-learning model and reduce the chances of missing flaws during deployment. Therefore, when curating a dataset for training deep-learning semantic segmentation models for digital X-ray radiography applications, an informed varying of the exposure conditions, as applied in this study, could yield a varied representation of flaws in terms of their CNR characteristics and potentially lead to a better generalization of the model.

## Figures and Tables

**Table 1:** Detailed summary of the exposure factors and the associated signal-to-noise ratio.

SN	Kilovoltage (kV)	Amperage (A)	Time (s)	Signal-to-Noise Ratio (SNR)
1	50 kV	100 µA	0.2	55–66
2	50 kV	100 µA	0.5	90–105
3	50 kV	200 µA	0.2	80–97
4	50 kV	200 µA	0.5	120–155
5	50 kV	450 µA	1	250–320
6	60 kV	100 µA	0.2	75–98
7	60 kV	100 µA	0.5	120–155
8	60 kV	200 µA	0.2	110–140
9	60 kV	200 µA	0.5	180–220
10	60 kV	400 µA	0.5	250–320
11	60 kV	450 µA	0.5	240–330
12	60 kV	450 µA	1	330–450
13	70 kV	100 µA	0.2	100–130
14	70 kV	100 µA	0.5	155–200
15	70 kV	200 µA	0.2	140–180
16	70 kV	200 µA	0.5	240–280
17	70 kV	400 µA	0.5	330–400
18	70 kV	450 µA	0.5	350–420
19	75 kV	450 µA	0.5	360–420
20	150 kV	50 µA	0.5	250–350

**Table 2:** A detailed description of the dataset preparation for four distinct datasets sorted according to CNR and SNR measurement values.

Group	Dataset Class	Range of Measurement Values for Dataset Class		Total no. of Sorted real Images	Data Class Split (% of Total) Train, Test, Validation (60%, 20%, 20%)	2nd Test Dataset (*test*_2_)	Range of Measurement Values of *test*_2_ Dataset	
1	High SNR	SNR	151–450	1408		Low SNR	SNR	51–150
2	Low SNR		51–300	1742		High SNR		301–450
3	High CNR	CNR	81–440	1243		Low CNR	CNR	1–20
4	Low CNR		1–80	1367		High CNR		301–440

**Table 3:** Mean intersection-over-union (IoU) values on 4 datasets sorted according to SNR and CNR values.

High SNR		Low SNR		High CNR		Low CNR	
test_1_ High SNR	*test*_2_ Low SNR	test_1_ Low SNR	*test*_2_ High SNR	test_1_ High CNR	*test*_2_ Low CNR	test_1_ Low CNR	*test*_2_ High CNR
0.9661	0.9683	0.9751	0.9609	0.9708	0.8305	0.9455	0.9701

**Table 4:** High CNR_2_ dataset sorted with reduced range of CNR measurement values.

Dataset Class	Range of Measurement Values for Dataset Class		Total no. of Sorted real Images	Data Class Split (% of Total) Train, Test, Validation (60%, 20%, 20%)	2nd Test Dataset (*test*_2_)	Range of CNR Measurement Values of *test*_2_ Dataset
High CNR_2_	CNR	201–440	312		Low CNR	1–20

**Table 5:** Mean intersection-over-union (IoU) values with High CNR_2_ datasets sorted according to CNR values with smaller range of measurement values.

High CNR_2_	
test_1_ High CNR	*test*_2_ Low CNR
0.9594	0.5875

## Data Availability

Data could be provided upon request through the corresponding author.
